# Polymer Conformations in Ionic Microgels in the Presence of Salt: Theoretical and Mesoscale Simulation Results

**DOI:** 10.3390/polym9010015

**Published:** 2017-01-05

**Authors:** Hideki Kobayashi, Rene Halver, Godehard Sutmann, Roland G. Winkler

**Affiliations:** 1 Theoretical Soft Matter and Biophysics, Institute of Complex Systems and Institute for Advanced Simulation, Forschungszentrum Jülich, D-52425 Jülich, Germany; kobayashih@mpip-mainz.mpg.de; 2 Jülich Supercomputing Centre, Institute for Advanced Simulation, Forschungszentrum Jülich, 52425 Jülich, Germany; r.halver@fz-juelich.de (R.H.); g.sutmann@fz-juelich.de (G.S.)

**Keywords:** microgel, nanogel, swelling, computer simulation, charge distribution, charge screening

## Abstract

We investigate the conformational properties of polymers in ionic microgels in the presence of salt ions by molecular dynamics simulations and analytical theory. A microgel particle consists of coarse-grained linear polymers, which are tetra-functionally crosslinked. Counterions and salt ions are taken into account explicitly, and charge-charge interactions are described by the Coulomb potential. By varying the charge interaction strength and salt concentration, we characterize the swelling of the polyelectrolytes and the charge distribution. In particular, we determine the amount of trapped mobile charges inside the microgel and the Debye screening length. Moreover, we analyze the polymer extension theoretically in terms of the tension blob model taking into account counterions and salt ions implicitly by the Debye–Hückel model. Our studies reveal a strong dependence of the amount of ions absorbed in the interior of the microgel on the electrostatic interaction strength, which is related to the degree of the gel swelling. This implies a dependence of the inverse Debye screening length *κ* on the ion concentration; we find a power-law increase of *κ* with the Coulomb interaction strength with the exponent 3/5 for a salt-free microgel and an exponent 1/2 for moderate salt concentrations. Additionally, the radial dependence of polymer conformations and ion distributions is addressed.

## 1. Introduction

Polyelectrolyte nano- and microgels are versatile polymer networks with a wide range of possible technical applications. In response to environmental stimuli, such as temperature, pH, the quality of solvent or as the ionic strength of the embedding fluid [1,2], microgels exhibit reversible volume changes. This renders them ideal candidates for a wide-range of applications in, e.g., template-based synthesis of inorganic nanoparticles, separation and purification technologies, sensing and drug delivery [3,4,5,6,7,8,9]. Moreover, polyelectrolyte gels are omnipresent in biological systems, e.g., the extra-cellular matrix or mucus [10,11]. Hence, a thorough understanding of the mechanisms that determine the structural features of a microgel is desirable for a rational design of novel functional materials. As a result, various theoretical studies have been performed to unravel the microgel swelling mechanisms [12,13,14,15,16,17,18,19,20]. A characteristic feature of nano- and microgels is their finite size and the presence of a gel-fluid interface. So far, the implications of the finite gel size on the microgel structural properties have hardly been analyzed, let alone has a theoretical description been provided [21,22,23,24,25,26]. As discussed in [26], we expect phenomena for microgels that are not present in macroscopic (bulk) systems. A particularly interesting aspect is the permeability of nano- and micro-gel particles for charged particles, e.g., counterions or salt ions. This permits a free exchange of ions between the microgel interior and its surroundings in response to conformational changes by environmental stimuli. As a consequence, typically, only a fraction of the ions necessary for local charge neutrality are arrested inside a microgel particle, in contrast to a bulk system, and a significant fraction is located in the neighborhood of a gel particle [21,23,26,27,28]. As a consequence, the microgel is typically not charge neutral. As is well known, strong Coulomb interactions lead to condensation of counterions and to their confinement in a gel particle. This is rather similar to the structures in macroscopic gels. Hence, the interplay between the counterion and salt ion distribution and the repulsion by the equally-charged monomers is fundamental for the structural features of nano- and microgels. Specifically, the non-neutrality has far reaching consequences for gel swelling.

There are various branched polyions, e.g., micelles, brushes and star polymers, that share various features with microgels, in particular permeability [27,29]. Still, geometric specificities imply distinct differences. Here, we mention the distribution of charges in star polymers, which is larger in the core part and decreases toward the periphery with respective distinct structural features [29].

As discussed in [26] for salt-free systems, microgels exhibit various swelling regimes depending on the Coulomb interaction strength and the distribution of counterions. For weak electrostatic interactions, the unscreened regime is obtained [29]. Here, a microgel swells due to strong repulsion of equally-charged monomers. At stronger Coulomb interactions, the screened regime appears [26], where screened electrostatic interactions (by counterions) determine the conformational properties of the polyelectrolytes. The microgel size in the various regimes can quantitatively be described by a mean-field expression based on an effective Debye–Hückel interaction between the monomers [26]. For strong Coulomb interactions, the microgel collapses due to counterion condensation [30].

In this article, we present results for the structural properties of microgels with and without added salt obtained by large-scale computer simulations, combining molecular dynamics simulations for the microgel particle with the Brownian multiparticle collision dynamics (B-MPC) method mimicking the uncorrelated background [31,32]. We are only interested in equilibrium properties and, thus, neglect hydrodynamic interactions. Charge-charge interactions are taken into account by the Coulomb potential, and counterions and salt ions are treated explicitly. We address the influence of salt on the microgel conformations in two ways, on the one hand, by simulations for various interaction strengths and over a certain range of salt concentrations and, on the other hand, by the scaling-type approach proposed in [26]. The latter allows for a systematic variation of the parameters, e.g., interaction strength and salt concentration, and the study of their effect on the microgel structure. We show that a quantitative agreement between simulation results and theoretical predictions requires accounting for the actual amount of counterions and salt ions confined in the microgel, which depends on the actual Coulomb interaction strength. Our studies provide insight into the charge-induced specific aspects of nano- and microgels and yield a hint at the crossover from the behavior of finite-size nanogel particles to large particles exhibiting macrogel behavior.

The article is organized as follows. In Section 2, we describe the applied microgel model and outline the simulation procedure. Section 3 presents a scaling consideration for the size of the microgel, which is compared with simulation results in Section 4. Further simulation results for the radial microgel properties are presented in Section 5, and Section 6 summarizes our findings.

## 2. Model

Our microgel particle consists of $N_{p}$ linear polyelectrolytes, which are linked by $N_{c}$ tetra-functional crosslinks [26]. An individual polyelectrolyte is modeled as a self-avoiding chain of $N_{m}$ coarse-grained monomers of mass *M* and charge $q_{m}=e$ [31,33]. Explicit counterions of the same size and mass as the monomers carry a charge $q_{c}=-e$. We consider an overall neutral system with an equal number of monomers and counterions. In addition, monovalent salt ions of charge ±e of concentration $c_{s}$ are taken into account. The initial structure of a microgel is obtained from an infinite diamond lattice structure with polymers connecting the lattice sites [17] by cutting off all polymers beyond a selected radius. This yields a polymer network in the interior of the remaining particle, as well as dangling ends at its surface. Although commonly used [15,26,34,35], this type of structure corresponds rather to a model network than a synthetic microgel. The latter comprises polydisperse polymers and an inhomogeneous crosslink density. Despite the difference, our approach will provide insight into the yet unexplored structure of microgels.

The bonds between monomers are described by the harmonic potential:

(1) $$U_{b}=\frac{\kappa_{b}}{2}\sum_{k}\left(\right|r_{k+1}-r_{k}{\left|-l\right)}^{2},$$

where *l* is the finite bond length and $\kappa_{b}$ is the strength of the bond potential [33]. Here, $r_{k}$ denotes the position of monomer *k*.

The finite size of the monomers and ions is taken into account by the truncated and shifted Lennard–Jones (LJ) potential [31]:

(2) $$\begin{array}{c} U_{\mathrm{LJ}}=\left\{\begin{array}{cc} 4\epsilon \left[{\left(\frac{\sigma }{r_{ij}}\right)}^{12}-{\left(\frac{\sigma }{r_{ij}}\right)}^{6}\right]-C_{c}, & r_{ij}<r_{c}\\ 0, & r_{ij}>r_{c}, \end{array}\right. \end{array}$$

with $r_{ij}=\left|r_{i}-r_{j}\right|$, $C_{c}=4\epsilon \left({\left(\sigma /r_{c}\right)}^{12}-{\left(\sigma /r_{c}\right)}^{6}\right)$ and $r_{c}$ the cut-off radius. This potential is often denoted as WCA (Weeks, Chandler, Andersen) potential [36]. The parameters *ϵ* and *σ* are the strength of the interaction and the diameter of the monomers and ions, respectively. We focus on microgels in a good solvent, i.e., we set $r_{c}=2^{1/6}\sigma$. Electrostatic interactions are captured by the Coulomb potential:

(3) $$U_{c}=\frac{1}{2}{\sum_{i,j}}^{\prime }\frac{q_{i}q_{j}}{\varepsilon |r_{i}-r_{j}|},$$

where *ε* is the dielectric constant of the implicit solvent, $q_{i}$ denotes the charge of the particle *i* and the prime indicates i≠j.

The microgel and ions are imbedded in a cubic simulation box, and periodic boundary conditions are applied. To capture the long-range Coulomb interactions, we employ the P2NFFT algorithm (Particle-Particle Nonequispaced Fast-Fourier Transform) [37,38]. The P2NFFT is part of the public ScaFaCoSlibrary [39] of scalable fast Coulomb solvers [40]. The velocity-Verlet algorithm [36] is applied to solve Newton’s equations of motion that govern the dynamics of the monomers and ions.

We perform isothermal simulations by combining the molecular dynamics simulations of monomers and ions with the Brownian multiparticle collisions dynamics approach (B-MPC) [31,32,41]. The latter is a variant of the multiparticle collision dynamics (MPC) simulation approach for fluids [31,42], where hydrodynamic interactions are switched off. In B-MPC, the monomer dynamics proceeds according to Newton’s equations, as described above. After a time interval Δt, denoted as collision time, an MPC collision is performed with a phantom particle. Thereby, we allocate a phantom particle to every monomer and ion with a mass *M* equal to the mass of a monomer and a velocity, which is taken from a Gaussian distribution with variance $Mk_{B}T$, where $k_{B}$ is the Boltzmann constant and *T* is the temperature. A collision consists of a rotation of a charged particle’s relative velocity, with respect to the center-of-mass velocity:

(4) $$v_{cm,i}=\frac{v_{i}+V}{2},$$

where $v_{i}$ is the velocity of monomer or ion *i* and V is the effective phantom particle velocity, around a randomly-orientated axis by a fixed angle *α*. The orientation of the rotation axis is chosen independently for every particle and every collision step. Hence, after a collision, the velocity of the *i*-th charged particle is:

(5) $$v_{i}\left(t+\Delta t\right)=v_{i}\left(t\right)+\left(R(\alpha )-I\right)\left(v_{i}-v_{cm,i}\right),$$

where R(α) is the rotation matrix and I the unit matrix [32,41].

The strength of the Coulomb interaction is characterized by the interaction parameter:

(6) $$\begin{array}{c} \Gamma =\frac{e^{2}}{\varepsilon lk_{B}T}=\frac{l_{B}}{l}\;, \end{array}$$

where *e* is the elementary charge and $l_{B}=e^{2}/\varepsilon k_{B}T$ is the Bjerrum length. Here, we study gel particles with $N_{p}=220$ and $N_{p}=1236$ polymers of length $N_{m}=20$. The respective numbers of crosslinks are $N_{c}=147$ and $N_{c}=729$. We represent length, energy and mass in units of *l*, $k_{B}T$ and *M*, respectively. The unit of time is $\tau =\sqrt{Ml^{2}/k_{B}T}$. The parameters of the Lennard–Jones potential Equation (2) are σ=0.8l and $\epsilon =k_{B}T$. To ensure nearly rigid bonds and to avoid bond stretching due to electrostatic interactions, we apply the spring constant $\kappa_{b}=10^{3}k_{B}T/l^{2}$, which ensures relative bond-length fluctuations below 1%.

## 3. Theoretical Estimation of Gel Size

**Figure 1:**
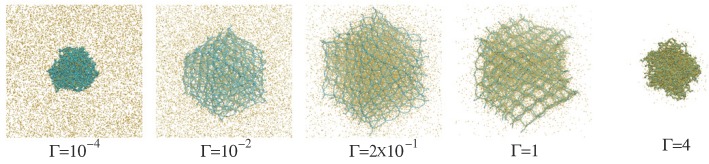
Snapshots of gel particles (blue) with $N_{c}=729$ crosslinks in the presence of counterions (yellow) for various Coulomb interactions strengths Γ as indicated.

As discussed in [26], microgel particles swell with increasing interaction strength Γ until counterion condensation sets in and the gel collapses. This is illustrated in Figure 1. The swelling of the microgel is accompanied by a stretching of the individual charged polymers. To reveal the dependence of the polymer stretching on the interaction strength and the concentration of ions and to characterize the stretching, we perform mean-field-type calculations applying the tension blob model [43]. We already discussed this approach in [26]. Here, we state the important steps and extend the description to microgels in the presence of salt.

In the tension blob model, a polymer is represented as a sequence of $N_{m}/g$ blobs, each comprising *g* monomers. Then, the average polymer extension is:

(7) $$\begin{array}{c} R_{E}\approx lg^{\nu }\frac{N_{m}}{g}, \end{array}$$

with ν≈0.6 for a good solvent. In the case of the microgel, due to the network structure, higher polymer and charge concentrations are present at the crosslinks. We assume that an individual polymer is stretched by the electrostatic forces between the blobs around the crosslinks at its ends. Since we consider tetra-functional networks, there are four blobs per chain ending in a crosslink. These interactions will exceed the forces by the other blobs along a polymer and practically govern the polymer elongation. This particularly applies for short polymers, as is typical for microgels. Hence, we can estimate the electrostatic energy by considering a gel particle as an aggregate of spherically-packed super blobs comprised of four blobs for every crosslink. The total Coulomb energy of the super blobs is proportional to the square of their charge $Q=4|e|g=4g\sqrt{\epsilon lk_{B}T\Gamma }$. To estimate the radius of the microgel in a scaling spirit, we write its total volume as $N_{p}R_{E}^{3}$, i.e., its radius is proportional to $N_{p}^{1/3}R_{E}$. The Coulomb energy is then proportional to $N_{c}^{2}Q^{2}/\epsilon N_{p}^{1/3}R_{E}$. This yields the total stretching energy:

(8) $$U_{s}=Ck_{B}T\frac{16N_{c}^{2}g^{2}\Gamma l}{N_{p}^{1/3}R_{E}}=N_{p}k_{B}T\frac{16g^{2}l\Gamma^{*}}{R_{E}},$$

with the effective interaction strength:

(9) $$\Gamma^{*}=C\frac{\Gamma N_{c}^{2}}{N_{p}^{4/3}}.$$

Here, we introduce the factor *C* as a free parameter accounting for particularities of the microgel structure. To account for present counterions and salt ions, we assume that the Coulomb interactions (8) are screened and describe them by the Debye–Hückel potential. The free energy of an individual stretched polyelectrolyte is then given by:

(10) $$\frac{F}{k_{B}T}=3\left(1-\nu \right){\left(\frac{R_{E}}{lN_{m}^{\nu }}\right)}^{1/(1-\nu )}+\frac{16g^{2}l\Gamma^{*}}{R_{E}}e^{-\kappa R_{E}},$$

where $\kappa =\sqrt{8\pi nl\Gamma^{*}}$ is the inverse Debye length involving the average density *n* of counterions and salt ions trapped in a microgel particle. The mean-field polymer end-to-end distance $R_{E}$ follows then from the condition $\partial F/\partial R_{E}=0$, where we will assume that *n* depends only weakly on $\Gamma^{*}$, or $R_{E}$ and, thus, can be neglected in the derivative of this term. As a limit, we obtain the scaling relation:

(11) $$\begin{array}{c} R_{E}∼R_{g}^{p}∼lN_{m}^{\left(2+\nu )/(4-\nu \right)}{\Gamma^{*}}^{\left(1-\nu )/(4-\nu \right)} \end{array}$$

for κ→0.

**Figure 2:**
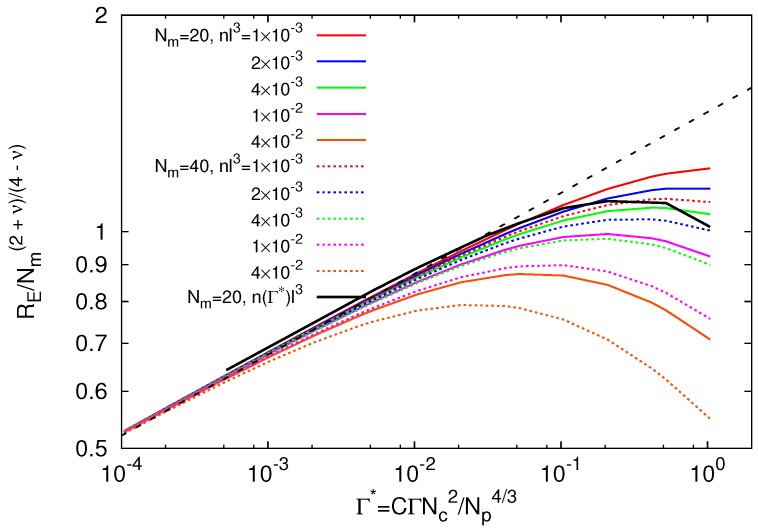
Theoretical prediction of the dependence of the polymer extension on the effective interaction strength $\Gamma^{*}$ for various ion concentrations *n* and the polymer lengths $N_{m}=20$ and 40. The thick black line is the simulation result for $N_{m}=20$, $N_{c}=147$ and L/l=200 [26].

Figure 2 shows results for $R_{E}$ following from the extremum of Equation (10). The various curves correspond to the indicated concentration of ions, which we consider here to be the same for all interaction strengths. Evidently, all curves approach the asymptotic behavior of Equation (11) for $\Gamma^{*}\to 0$, with the power-law dependence $R_{E}∼{\Gamma^{*}}^{2/17}$ for the critical exponent ν=3/5 in agreement with simulations [26]. Our exponent (1−ν)/(4−ν)≈0.12 (ν=3/5) is obviously smaller than that of an isolated polyelectrolyte chain [43,44]. Hence, the properties of a polyelectrolyte part of a microgel are rather different from a free polyelectrolyte, i.e., crosslinks play a crucial role in microgel swelling.

Screening of the monomer-monomer electrostatic interactions leads to a saturation of the polymer extension and, for large ion concentrations, a shrinkage of a polymer and, consequently, the microgel. Thereby, the maximum extension depends on *n*, and the maximum of $R_{E}$ shifts to smaller values with increasing ion concentration. Nevertheless, the individual curves exhibit a plateau-like regime over approximately a decade of interaction strengths. Comparing the two considered polymer lengths, we find a seemingly stronger screening effect for the longer polymer ($N_{m}=40$). However, this is simply related to the chosen normalization factor $lN_{m}^{\left(2+\nu )/(4-\nu \right)}$. According to Equation (11), the end-to-end distance is larger for longer polymers. Since the Debye–Hückel interaction dominates the free energy for large $\Gamma^{*}$, the length-dependence becomes weaker than the factor of Equation (11).

So far, we considered an assembly of point-like blobs and ions screening the charge interactions. However, assuming finite size blobs, ions are not only distributed between blobs, but also penetrate the blobs and lead to a reduction of the effective charge relevant for our free energy Equation (10). With the volume $V_{b}=4\pi g^{3\nu }/3$ of a blob and the ion density *n*, the total number of ions in a blob can be approximated as $nV_{b}$, which implies the effective charge of a blob $\left|e|(4g-nV_{b}\right)$. Hence, the free energy becomes:

(12) $$\frac{F}{k_{B}T}=3\left(1-\nu \right){\left(\frac{R_{E}}{lN_{m}^{\nu }}\right)}^{1/(1-\nu )}+\frac{{\left(4g-nV_{b}\right)}^{2}l\Gamma^{*}}{R_{E}}e^{-\kappa R_{E}}.$$

Now, we again consider point-like blobs, but with an effective (smaller) charge. This modification disappears for salt-free systems, since the concentration *n* becomes small.

## 4. Simulation Results: Comparison with Analytical Theory

Figure 2 indicates a strong dependence of the microgel conformations on the concentration of ions. To quantify the counterion and salt ion contributions to screening for the various interactions strengths, we determine the ion density inside a microgel and calculate the screening length *κ*. Thereby, we define the ion density as $n=N_{ion}/V_{g}$, where $N_{ion}$ is the number of ions inside the sphere of radius $3R_{g}/2$, where $R_{g}$ is the interaction strength-dependent radius of gyration of the microgel [26] and $V_{g}=4\pi {\left(3R_{g}/2\right)}^{3}/3$ is the respective gel volume.

### 4.1. Salt-Free Microgel

**Figure 3:**
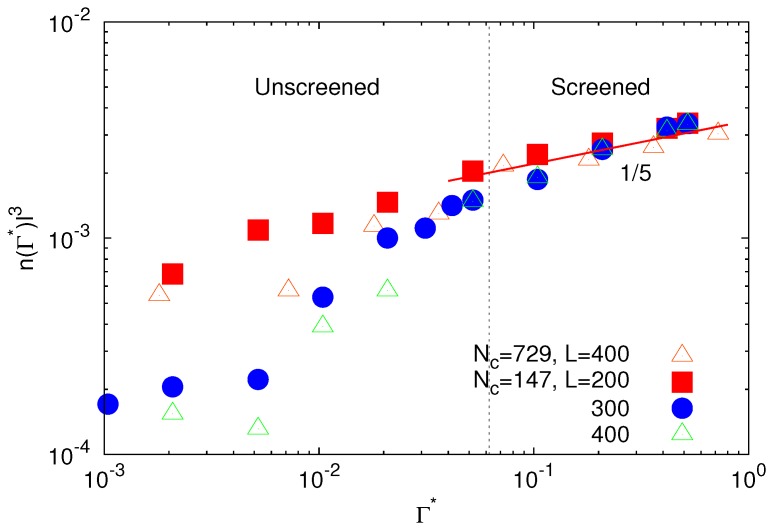
Density of counterions inside a microgel as a function of the scaled interaction strength for microgels with $N_{c}=147$ and 729 crosslinks. For the larger microgel, the size of the simulation box is L/l=400, and for the smaller one with $N_{c}=147$, the boxes are L/l=200, 300 and 400.

The counterion densities for the two gel sizes $N_{c}=147$ and 729 and the various system sizes (microgel concentrations) are displayed in Figure 3. As expected, the counterion density inside the microgel increases with increasing interaction strength. Interestingly, this increase depends only weakly on the gel and box size for $\Gamma^{*}>5\times 10^{-2}$, where $n(\Gamma^{*})$ exhibits the power-law dependence ${\Gamma^{*}}^{\gamma }$, with γ≈1/5. This weak dependence of *n* on $\Gamma^{*}$ justifies our assumption of a nearly constant *n* in the calculation of the derivation of Equation (10) with respect to $R_{E}$. For $\Gamma^{*}<5\times 10^{-2}$, we observe a significant dependence of the counterion concentration on the interaction strength. However, the Coulomb interaction is here very weak and counterions play a minor role for the microgel conformational properties [26]. Only for larger $\Gamma^{*}$, electrostatics matters. This is particularly evident for the largest system with L/l=400 and the smaller microgel. Here, the counterion density increases rapidly in the range $10^{-2}<\Gamma^{*}<5\times 10^{-2}$ and approaches a rather similar asymptotic behavior as for the smaller systems. Hence, Coulomb interactions start to become essential for $\Gamma^{*}≳5\times 10^{-2}$. This is consistent with our previous findings and our definition of the unscreened and screened regimes of electrostatic interactions [26].

**Figure 4:**
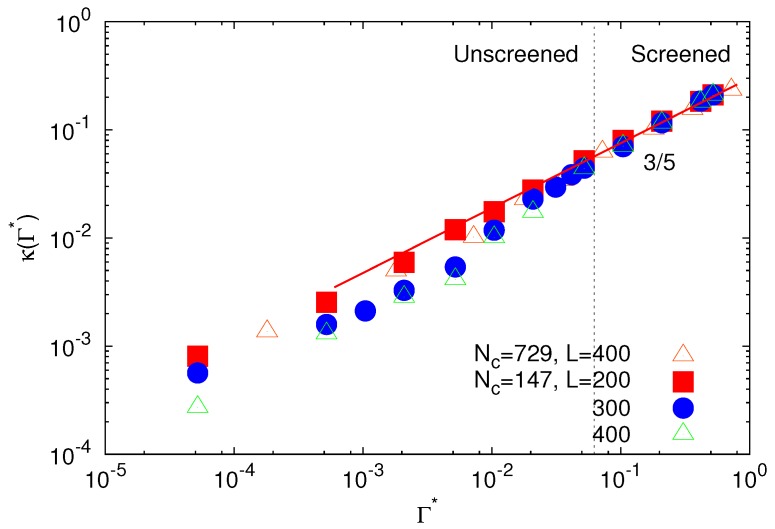
Inverse Debye screening length as a function of the scaled interaction strength for microgels with $N_{c}=147$ and 729 crosslinks. For the larger microgel, the size of the simulation box is L/l=400, and for the smaller one with $N_{c}=147$, the boxes are L/l=200, 300 and 400.

Naturally, the interaction strength-dependent counterion density leads to a $\Gamma^{*}$-dependent Debye screening length. The respective dependence of $\kappa =\sqrt{8\pi nl\Gamma^{*}}$ on $\Gamma^{*}$ is presented in Figure 4. Consistent with the exponent *γ*, *κ* exhibits a power-law increase with increasing interaction strength with the approximate exponent γ≈3/5 in the screened regime, independent of microgel size and density. Only in the unscreened regime, we find small *κ* values for the lower microgel densities.

Simulation results [26] for the polymer size are presented by the black solid line in Figure 2. As shown in [26], the simulation results are very well described by the theoretical expression. In Figure 2, the simulation data are only in qualitative agreement with the theoretical result. The precise shape of the curves depends on the actual, Γ-dependent amount of counterions inside the microgel particle. In fact, it is important to account for the actual counterion concentration rather than its change, as confirmed by the agreement achieved in Figure 1 of [26].

### 4.2. Microgel in Presence of Salt

**Figure 5:**
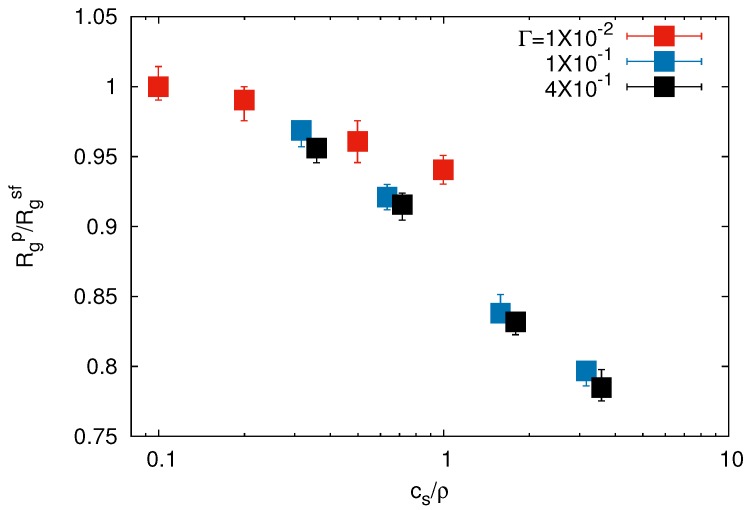
Dependence of the average radius of gyration of individual polymers $R_{g}^{p}$ on the ratio of the salt concentration $c_{s}$ and network charge density *ρ* for $N_{c}=147$ and L/l=200. $R_{g}^{sf}$ is the polymer radius of gyration of the salt-free system.

The influence of salt on the conformational properties of the polymers is illustrated in Figure 5. We add $N_{s}=10^{4}-10^{5}$ monovalent salt ions of charges ±e, such that the overall system remains neutral. As for the monomers and counterions, the strength of the electrostatic interaction is characterized by Γ of Equation (6). Figure 5 shows average radii of gyration $R_{g}^{p}$ of individual polymers as a function of $c_{s}/\rho$, where $c_{s}=N_{s}/V$ is the salt concentration and $\rho =N/V_{g}$ the network monomer density. The size of a gel particle is hardly affected by the presence of salt ions as long as $c_{s}/\rho ≲0.4$ for any chosen Γ. For concentrations $c_{s}/\rho ≳0.6$, $R_{g}^{p}$ decreases with increasing salt content. Our results are in good agreement with previous studies. For micrometer-size gels, theoretical investigations suggest that the size of a gel particle is roughly constant with increasing salt content until $c_{s}$ becomes comparable with *ρ* [45]. Only when $c_{s}$ exceeds a certain value, a gel particle exhibits strong shrinkage. This prediction has been confirmed by various experiments [46,47,48] and numerical studies [49]. Even for nanometer-size gels, numerical studies [25] reported that the gel radius shows only minor changes upon an increase of the concentration of monovalent salt.

**Figure 6:**
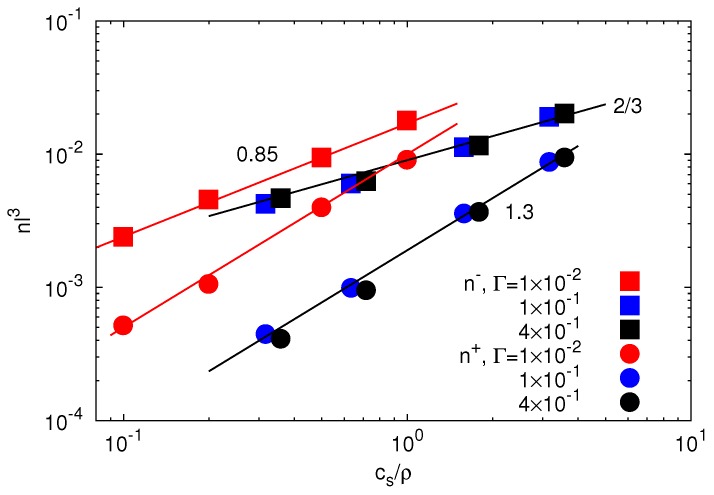
Density of ions inside a microgel as a function of the salt concentration for microgels with $N_{c}=147$ and L/l=200 and the indicated interaction strengths. The squares represent the total concentration of negatively-charged ions, i.e., counterions plus salt ions. The bullets represent the positively-charged salt ions.

The concentration of ions inside a microgel as a function of the salt concentration is shown in Figure 6. The total number of negative ions, i.e., counterions plus respective salt ions, increases approximately in a power-law fashion in the considered range of salt concentrations. Thereby, the exponent depends on the Coulomb interaction strength. For smaller Γ ($\Gamma =10^{-2}$), *n* increases with an exponent of approximately 0.85. For larger Γ, the exponent is approximately 2/3. In any case, the concentration of ions in the presence of salt is higher. For $\Gamma =10^{-2}$, the ratio $n/n^{sf}$, where $n^{sf}$ is the counterion concentration in the salt-free microgel, increases by $n/n^{sf}\approx 2-10$. This increase appears rather large, but it simply reflects the linear increase in the salt ion concentration and their homogeneous distribution over the available volume. With increasing Γ, the ratio $n/n^{sf}$ decreases, because, on the one hand, the counterion concentration is higher, and on the other hand, the counterions contained in the microgel neutralize part of the microgel charge. The ratio itself changes by $n/n^{sf}\approx 1.5-3$ over the considered range of salt concentrations. Thereby, $n/n^{sf}$ increases in a power-law manner with increasing salt concentration with the approximate exponents 0.85, for $\Gamma =10^{-2}$, and 2/3, for $\Gamma =10^{-1}$ and $4\times 10^{-1}$. The significantly higher counterion concentration inside the microgel compared to the surroundings (cf. Figure 2 of [26]) prevents evidently an even spread of the salt ions.

Figure 6 shows that the concentration of salt coions $n^{+}$ inside the microgel is always smaller than that of *n*, but the concentration increases substantially faster than *n* with increasing salt concentration. This gives rise to an additional screening of the electrostatic interactions and prevents a charge inversion of the whole microgel.

**Figure 7:**
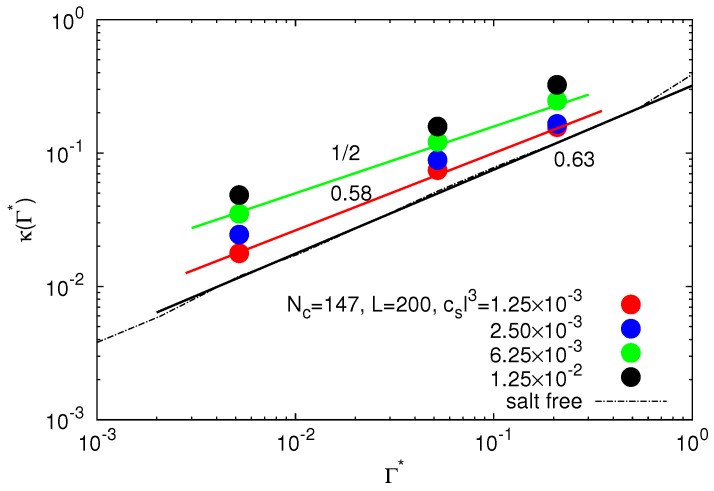
Inverse Debye screening length as a function of the scaled interaction strength for microgels with $N_{c}=147$ crosslinks, L/l=200 and various salt concentrations. The dashed line represents the salt-free system.

The dependence of the Debye screening length on the effective interaction strength is shown in Figure 7. Clearly, the inverse Debye length increases with increasing salt concentration. For small interaction strengths ($\Gamma <10^{-2}$), *κ* increases by the same power-law as *κ* of the salt-free case. However, for a larger salt content, we find the approximate dependence $\kappa ∼{\Gamma^{*}}^{1/2}$. Hence, the inverse Debye length increases only slowly with the Coulomb interaction strength, following essentially the definition $\kappa =\sqrt{8\pi nl\Gamma^{*}}$ with a concentration independent *n*. This implies that our analytical predictions for the polymer swelling in Figure 2 should very well describe the polymer properties for $c_{s}l^{3}≳6.25\times 10^{-3}$.

Finally, we would like to link our salt studies to recent simulation results presented in [50]. In [50], the concentration dependence of the excess chemical potential of ion pairs has been determined as a function of the ratio of the Bjerrum length and the ion radius. The calculations yield a pronounced dependence of the chemical potential on that ratio for larger salt concentrations, which suggests that electrolyte solutions have to be carefully modeled in order to avoid artifacts by inappropriate choices of particle sizes. Comparing our parameters and salt concentrations with the predictions of [50], our concentrations are in the range $c_{s}<10^{-3}$mol/L of [50] for all interaction strengths as long as $c_{s}/\rho <1$. Hence, we are in the regime, where agreement between theory and simulations is obtained [50]. Since our theoretical considerations of Section 3 employ the same definitions and concentrations, they are also applicable to solutions with monovalent salt and salt concentrations up to approximately 40% of the network charge of the gel particle.

**Figure 8:**
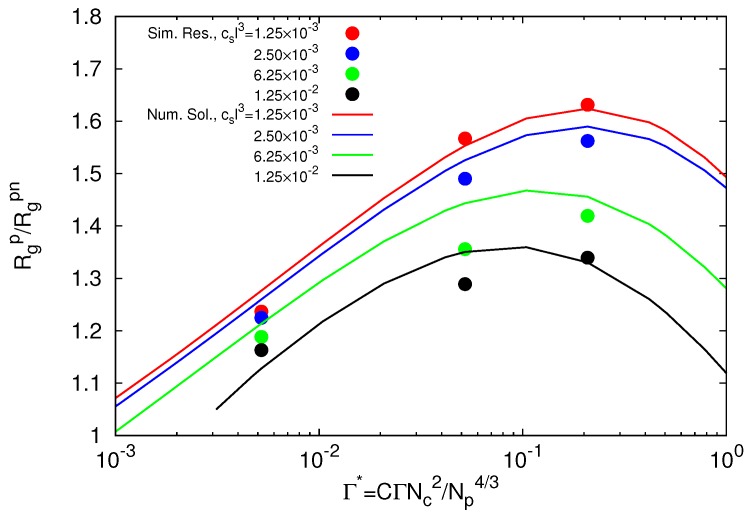
Average radii of gyration $R_{g}^{p}$ of individual polymers as a function of the interaction strength $\Gamma^{*}$ for microgels with $N_{c}=147$ and various salt concentrations $c_{s}$. The average radius of gyration of the neutral system is $R_{g}^{pn}/l=2.6$. The theoretical curves follow for the parameters C=0.032 and $\hat{C}=1.5$.

With the densities of ions in a microgel and the inverse screening length, Figure 6 and Figure 7, we are able to validate our theoretical prediction for the polymer swelling, following the derivative of the free energy Equation (12), against simulation results. With the given charge concentration and screening length, there is only one fitting parameter, namely *C* in Equation (9). To account for additional possible discrepancies between our simple theoretical approach and simulation results, we introduce a further fitting parameter $\hat{C}$ by replacing the average charge concentration *n* by $\hat{C}n$. The simulation results and fitted theoretical curves are compared in Figure 8. The fit parameters are C=0.032 and $\hat{C}=1.5$. The latter corresponds to an increase of the inverse Debye screening length by a factor of 1.22. Evidently, the theoretical expression captures the dependence of the polymer swelling on the interaction strength and salt concentration well over the considered and experimentally-relevant ranges. The parameter $\hat{C}$ is astonishingly close to unity. Various effects might be responsible for a $\hat{C}>1$, such as an inhomogeneous dielectric constant or specificities in the inhomogeneous charge distribution. In any case, our approach captures the essence of the underlying physical mechanisms well, particularly when considering its simplicity.

## 5. Simulation Results: Radial Microgel Properties

The finite size of a microgel implies radial variations of the polyelectrolyte and counterion properties. Here, we discuss a variety of these quantities and extract universal properties between microgels of different sizes.

### 5.1. Radial Monomer Distribution

**Figure 9:**
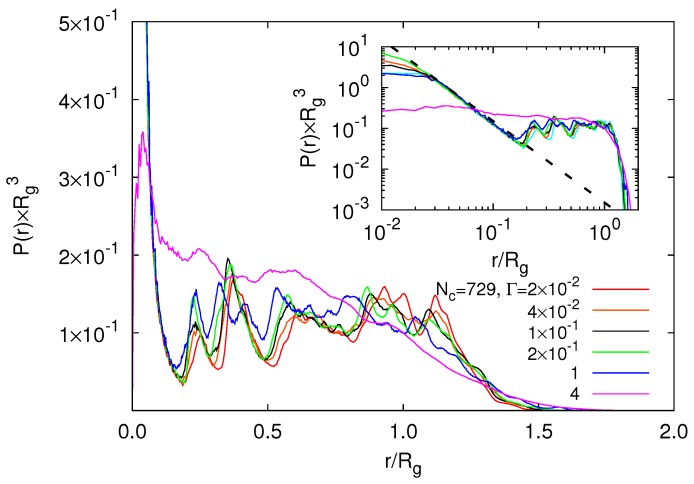
Normalized radial distribution functions P(r) of monomers, with respect to the microgel center of mass, for various interaction strengths of nanogels with $N_{c}=729$ and L/l=400. The inset shows the distribution function on a log-log scale. The dashed lines is proportional to $r^{-2}$. The radii of gyration of the microgels are $R_{g}/l=75.1,\;77.1,\;81.7,\;82.1,\;78.2$ and 40.2 with increasing Γ.

The radial modulation of the monomer concentration is reflected in Figure 9. The radial distribution function P(r) exhibits characteristic peaks due to the underlying diamond-lattice structure. Thereby, the distributions depend only weakly on Γ in the range $2\times 10^{-2}<\Gamma <1.0$. Only for Γ≳4, a rather homogeneous structure is achieved due to the gel collapse. P(r) decreases as $r^{-2}$ with increasing radial distance for $r/R_{g}<0.2$ as shown in the inset of Figure 9. This reflects the topological structure of our system with one tetra-functional crosslink close to the center of mass. This is similar to the behavior of star polymers [51]. For Γ>0.01, we find a monomer depletion zone in the vicinity of $r/R_{g}\approx 0.2$ and an enhanced density at $r/R_{g}\approx 0.7$. This indicates that peripheral polymers are more compact than internal ones. A similar behavior has been found for swollen states of a microgel, where monomer interactions are described bye the Debye–Hückel potential [34]. From the distribution function, we anticipate that a weakly modulated radial regime of nearly-constant monomer density will appear for even larger microgel particles. Nevertheless, there remains a depletion zone near the center of the microgel.

### 5.2. Radial Polymer Conformations

**Figure 10:**
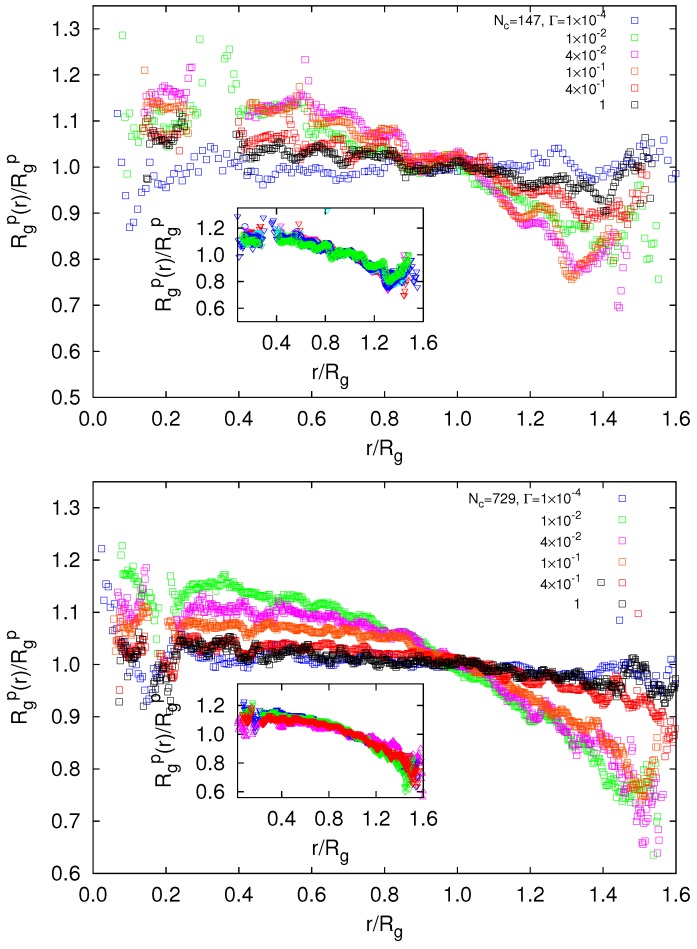
Dependence of the polymer radius of gyration on its radial center-of-mass position *r* for (top) $N_{c}=147$ and L/l=300, and (bottom) for $N_{c}=729$ and L/l=400. The insets show radii of gyration in the unscreened regime. The radii of gyration of the microgels are (top) $R_{g}/l=17.9,\;31.7,\;40.5,\;44.6,\;45.7$ and 44.0, and (bottom) $R_{g}/l=33.9,\;67.7,\;77.1,\;81.7,\;80.7$ and 40.2 with increasing Γ.

As shown in Figure 2, the average radius of gyration of the polymers depends on the interaction strength. The radial dependence of $R_{g}^{p}$ itself is illustrated in Figure 10. As is evident from the figure, the dependence of $R_{g}^{p}\left(r\right)$ on the electrostatic interaction is qualitatively the same for both gel sizes. For $\Gamma ≲10^{-4}$, the polymer conformations are nearly homogeneous across the gel, aside from modulations close to the center of mass. As soon as the gel swells, $R_{g}^{p}\left(r\right)$ of the polymers in the inner part ($r/R_{g}<1$) swells stronger than those in the periphery. This tendency applies for interaction strengths within the unscreened regime (cf. Figure 2). This is emphasized in the insets of Figure 10. Hence, nano- and microgels exhibit a strong inhomogeneous structure as a consequence of their finite size. For the interaction strengths Γ≳0.2 ($N_{c}=147$) and Γ≳0.1 ($N_{c}=729$), the polymers at $r/R_{g}>1$ swell again, and those in the central part simultaneously shrink (the screened regime in Figure 2). We attribute this behavior to screening of monomer electrostatic interactions by counterions. Consequently, microgels show a homogeneous structure again in terms of polymer swelling at large interaction strengths. The ratio of the maximum and minimum values of $R_{g}^{p}\left(r\right)$ extends up to approximately 1.6. This is clearly larger than the ratio obtained by the calculation with a Debye–Hückel interaction [34]. These features are a consequence of the finite size of nano- and microgels and are not observed in bulk systems.

**Figure 11:**
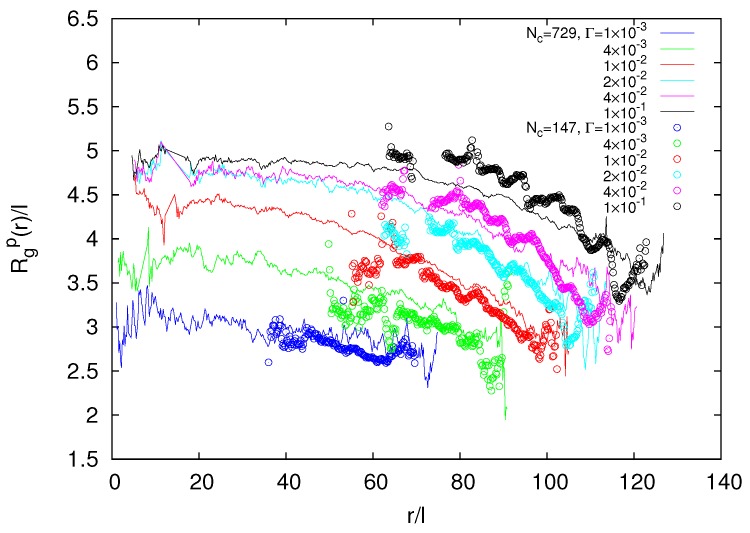
Dependence of the polymer radius of gyration on its radial center-of-mass position *r* for microgels with $N_{c}=729$ and L/l=400 (lines), and $N_{c}=147$ and L/l=300 (circles). The latter are radially shifted to match the respective microgel surface.

Interestingly, the radii of gyration of the smaller gel closely agree with those of the larger gel in their periphery for Γ<0.1, i.e., when we shift the radial coordinate *r* of the gel with $N_{c}=147$ to match the surface of the larger gel, as indicated in Figure 11. This implies that $R_{g}^{p}\left(r\right)$ is determined by the distance from the surface of the gel particle. The superposition of the curves helps us to shed light on the crossover from nano- and micro-gel to macrogel properties. Evidently, our smaller gel particle is completely determined by finite size effects, whereas the larger one exhibits the same structure in the periphery, but assumes already a more macrogel-like behavior in the central region. This crossover to macrogel behavior is also visible in Figure 9.

**Figure 12:**
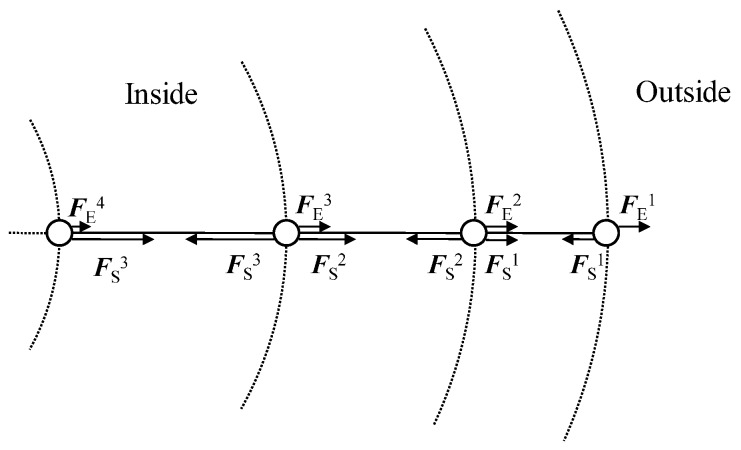
Schematic illustration of the force balance on the nanogel network. Circles and solid lines express crosslinks and individual polymers, respectively.

To illustrate the stronger stretching of polymers toward the center of the gel particle, we consider a radial structure of “crosslinks” as depicted in Figure 12. The crosslinks of the individual polymers are aligned in a straight line. We assume that individual polymers are stretched by electrostatic force $F_{i}^{E}$ between crosslinks, which are counterbalanced by entropic forces $F_{i}^{S}$ of polymers. Here, *i* enumerates the crosslinks from the shell toward the core. In steady state, the force balance at each crosslink requires:

(13) $$\begin{array}{ccc} F_{1}^{S} & = & F_{1}^{E}, \end{array}$$

(14) $$\begin{array}{ccc} F_{2}^{S} & = & F_{2}^{E}+F_{1}^{S}, \end{array}$$

(15) $$\begin{array}{ccc} F_{i+1}^{S} & = & F_{i+1}^{E}+F_{i}^{S}. \end{array}$$

Equation (15) implies $F_{i+1}^{S}\geq F_{i}^{S}$; the forces are only equal when $F_{i}^{E}=0$. The elongation of individual polymers is directly reflected in $F_{i}^{S}$. Thus, the relation $F_{i+1}^{S}\geq F_{i}^{S}$ implies that inside polymers are more stretched than outside ones. After recovering electrostatic neutrality and a homogeneous distribution of counterions, $F_{i}^{E}=0$, and $R_{g}^{p}\left(r\right)$ is essentially constant. Although our consideration is rather crude, the polymer properties of Figure 10 are consistent with it. Moreover, $R_{g}^{p}\left(r\right)$ exhibits a plateau for $r/R_{g}\leq 0.8$ and Γ≥0.1, as shown in Figure 10. This bulk-like behavior implies the existence of a crossover from nano- to macrogels.

### 5.3. Radial Counterion Distribution

**Figure 13:**
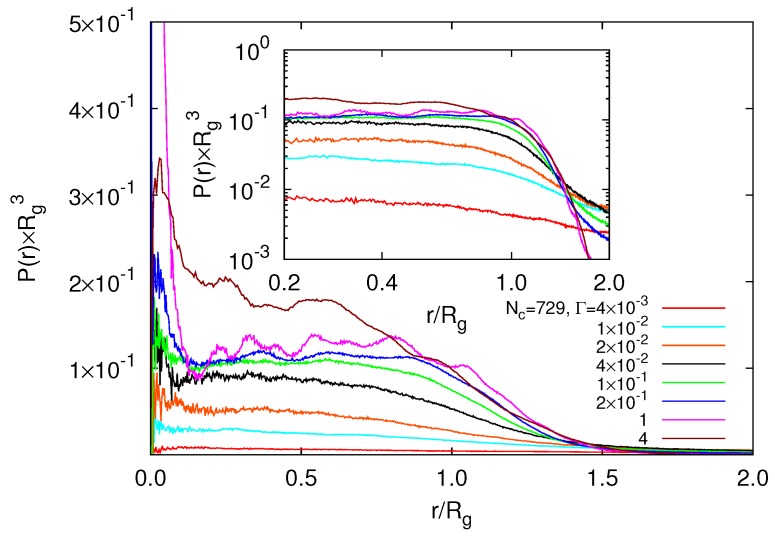
Normalized radial counterion distribution functions P(r), with respect to the microgel center of mass, for various interactions strengths and the microgel of size $N_{c}=729$ and L/l=400. The inset shows the same quantities in a semilogarithmic representation. The radii of gyration of the microgels are $R_{g}/l=$ 56.7, 67.7, 75.1, 77.1, 81.7, 78.2 and 40.2 with increasing Γ.

Figure 13 shows radial distribution functions of counterions for various interaction strengths. In the nearly neutral systems $\Gamma ≲10^{-4}$, the counterion density is essentially homogeneous over the whole periodic system, i.e., it is approximately the same inside and outside of the gel particle. With increasing Coulomb interaction strength, the density of counterions within a gel particle ($r/R_{g}<1.5$) increases at the expense of the outside ions. For large Γ, the attractive Coulomb interaction between charged monomers and counterions effectively confines a major fraction of counterions inside of the gel. However, the density of counterions remains lower than those of monomers for Γ≲1. Above Γ≈1, counterion condensation sets in, and the ion distribution reflects the monomer distribution. In the limit Γ≫1, essentially all counterions are condensed.

### 5.4. Radial Effective Charge

As discussed in Section 3, the absorption of counterions implies a screening of electrostatic interactions inside of a gel particle. In order to characterize the net charge, specifically its radial dependence, we introduce the net radial charge $I_{m}\left(r\right)-I_{i}\left(r\right)$, where $I_{m/i}$ are the integrals of the monomer or counterion density, respectively, i.e.,

(16) $$\begin{array}{c} I_{m/i}\left(r\right)=4\pi \int_{0}^{r}P_{m/i}\left(r^{\prime }\right)r^{\prime 2}dr^{\prime }\;. \end{array}$$

**Figure 14:**
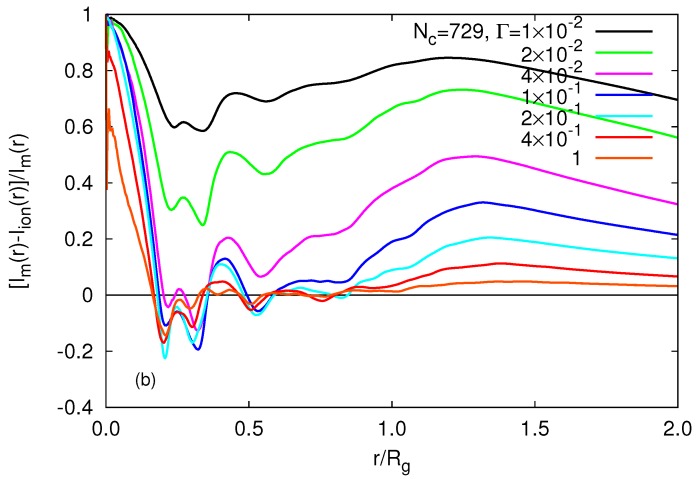
Ratio of the monomer-counterion charge difference and the monomer charge (cf. Equation (16)) as a function of the radial distance from the microgel center for the indicated interaction strengths and the microgel of size $N_{c}=729$ with L/l=400. The radii of gyration of the microgels are $R_{g}/l=$ 67.7, 75.1, 77.1, 81.7, 82.2, 80.7 and 78.2 with increasing Γ.

Ratios of the net radial charge and the radial monomer charge are displayed in Figure 14. By comparing Figure 14 with the respective charge distribution for the smaller microgel presented in [26], the ratio evidently depends on the system size, similar to other physical properties related to the counterion concentration. The ratio $I_{m}\left(r\right)/I_{i}\left(r\right)$ is always positive in the unscreened regime, independent of $N_{c}$. Hence, the monomer charges dominate over the counterion charges, and the gel interior is oppositely charged compared to the counterions. For 0.04≲Γ≲1, the counterion charge dominates over the monomer charge at certain radii inside of the gel, specifically in the vicinity of $r/R_{g}\approx 0.25$. Beyond $r/R_{g}\approx 1$, the net charge is nonzero and always of the same sign as the monomer charge. Note that the shape of the radial net charge naturally depends on the network structure of the gel particle. Since the gel extends up to $r/R_{g}\approx 1.5$ (cf. Figure 9 and Figure 13), a remaining charged volume surrounds the interior screened volume, i.e., the ratio of the net-charged volume to the whole gel-particle volume is sufficiently high to yield an effective net charge. Interestingly, the counterion charge approximately balances the monomer charge over a rather extended region $0.5≲r/R_{g}≲1$ for $4\times 10^{-2}≲\Gamma ≲1$. This large range of a nearly neutral radial shell marks the crossover from the behavior of a nano- and micro-gel to a macroscopic gel. For a macrogel, we expect only a relatively thin charged surface layer; the rest of the gel should be neutral. The gel reflects this behavior above $\Gamma \approx 4\times 10^{-2}$.

### 5.5. Monomer-Counterion Pair Distribution Function

The local arrangement of the counterions with respect to the monomers is captured in the monomer-counterion distribution function $g_{mi}\left(r\right)$. We define this distribution as:

(17) $$\begin{array}{c} 4\pi \int g_{mi}\left(r\right)r^{2}dr=\frac{NV_{g}}{N_{i}}. \end{array}$$

**Figure 15:**
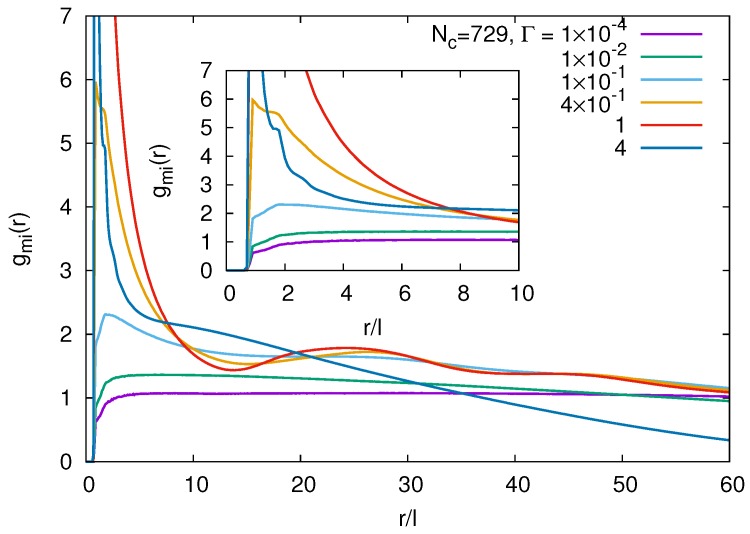
Monomer-counterion pair correlation functions for various interaction strengths Γ. The number of crosslinks is $N_{c}=729$, and the box size L/l=400. The inset shows the correlation functions over a smaller range r/l.

Figure 15 shows correlation functions for the larger gel. Those of the smaller gel particle are rather similar and are thus not discussed. For r/l<1, the correlation function is zero due to the finite size of monomers and counterions. At small interaction strengths $\Gamma <10^{-2}$, we find essentially a constant, gas-like distribution of ions with small modulations in the range 1<r/l<2. The latter behavior is understood from Equation (17) and is independent of *N* and *V*. A similar structure has been obtained analytically for solutions of rod-like polyelectrolytes in [52] by an integral equation theory approach. With increasing interaction strength, a strong peak develops at the nearest neighbor distance r/l≈1. Since the number of ions is limited, the correlation function strongly drops below unity at large distances. In the screened regime (cf. Figure 2), strong second and third neighbor peaks appear, indicating strong correlation between monomers and counterions. The counterions are not strongly bound to monomers yet, but preferentially align along the polymers (cf. Figure 1). Additional peaks develop for $\Gamma ≳4\times 10^{-1}$ at r/l≈25 and r/l≈45, or $r/R_{g}\approx 0.3$ and $r/R_{g}\approx 0.6$. They are related to the particular structure of the underlying lattice and correspond to the strong radial monomer and counterions peaks of Figure 9 and Figure 13 at the respective distances. Large interaction strengths (Γ≳4) lead to counterion condensation, and the pair correlation function assumes rather large values over a wide range of monomer-counterion distances corresponding to the dense packing and a seemingly homogeneous distribution of charges.

## 6. Summary and Conclusions

We have presented simulation results for the structural properties of finite-size crosslinked polyelectrolyte networks with and without added salt. Thereby, we have addressed the polymer properties, as well as those of the explicit ions. Moreover, we have analyzed the polymer swelling theoretically applying the tension blob model taking into account counterions and salt ions implicitly by the Debye–Hückel model. Our studies provide detailed insight into the swelling behavior of finite-sized ionic gel particles. Most importantly, in the unscreened regime $4\times 10^{-4}<\Gamma^{*}<5\times 10^{-2}$, Coulomb interactions drive the swelling of individual polymers and networks. Here, counterions play a minor role. Counterions start to screen the Coulomb interactions in the screened regime $5\times 10^{-2}<\Gamma^{*}<CN_{c}^{2}/N_{p}^{4/3}$, and the polymer conformations only weakly depend on the electrostatic interaction, which is quantitatively described by Equations (10) and (12). Our studies reveal an increase of the ion concentration *n*, i.e., the concentration of counterions and equally-charged salt ions, inside the microgel with increasing salt concentration. For the considered range of (moderate) salt concentrations, we find a power-law dependence for *n* on the interaction strength $\Gamma^{*}$ (cf. Figure 6). As a consequence, the inverse Debye screening length *κ* increases as $\kappa ∼{\Gamma^{*}}^{3/5}$ at large $\Gamma^{*}$ and small salt concentrations. Interestingly, we recover the dependence $\kappa ∼{\Gamma^{*}}^{1/2}$ for higher salt concentrations. This in turn implies a very weak dependence of the ion concentration on the interaction strength in that regime. Taking into account a reduction of the effective blob charge by present salt ions, our mean-field approach captures the polymer swelling very well also in the case of salt.

In the unscreened regime, the electrostatic neutrality of the gel particle and the absorbed counterions is violated. Thereby, radially inhomogeneous polymer conformations appear prominently. In contrast, in the screened regime, the electrostatic neutrality is recovered in certain volumes around the center-of-mass of a microgel. Thereby, this volume strongly depends on the gel size and increases with increasing gel size. This might be expected, since very large gel particles should exhibit properties of macrogels over a large volume, and only a peripheral shell should remain charged. This indicates a crossover from microgel to macrogel behavior above a certain particle size.

Considering the local polymer conformations, we find significantly stronger stretched polymers in the core of a gel particle ($r/R_{g}<1$) and only weakly stretched ones in the periphery in the unscreened regime. As soon as a significant amount of counterions is absorbed by the gel, the polymers assume homogeneous conformations.

Our studies reveal rich structural properties of microgel particles due to their finite size. Various aspects, such as a lower density in the microgel core and a higher one in the shell, can be exploited in the quest for functional materials, e.g., the inclusion of guest particles and molecules. Hence, we hope that our studies are helpful in future experimental endeavors to synthesize novel functional microgel particles.
